# Environmental Enrichment Reduces Anxiety by Differentially Activating Serotonergic and Neuropeptide Y (NPY)-Ergic System in Indian Field Mouse (*Mus booduga*): An Animal Model of Post-Traumatic Stress Disorder

**DOI:** 10.1371/journal.pone.0127945

**Published:** 2015-05-27

**Authors:** Durairaj Ragu Varman, Koilmani Emmanuvel Rajan

**Affiliations:** Department of Animal Science, School of Life Sciences, Bharathidasan University, Palkalaiperur campus, Tiruchirappalli, India; University of Insubria, ITALY

## Abstract

Exposure to a predator elicits an innate fear response and mimics several behavioral disorders related to post-traumatic stress disorder (PTSD). The protective role of an enriched condition (EC) against psychogenic stressors in various animal models has been well documented. However, this condition has not been tested in field mice in the context of PTSD. In this study, we show that field mice (*Mus booduga*) housed under EC exhibit predominantly proactive and less reactive behavior compared with mice housed under standard conditions (SC) during exposure to their natural predator (field rat *Rattus rattus*). Furthermore, we observed that EC mice displayed less anxiety-like behavior in an elevated plus maze (EPM) and light/dark-box after exposure to the predator (7 hrs/7 days). In EC mice, predator exposure elevated the level of serotonin (5-Hydroxytrypamine, [5-HT]) in the amygdala as part of the coping response. Subsequently, the serotonin transporter (SERT) and 5-HT_1A_ receptor were up-regulated significantly, but the same did not occur in the 5-HT_2C_ receptor, which is associated with the activation of calmodulin-dependent protein kinase-II (CaMKII) and a transcription factor cAMP response element binding protein (CREB). Our results show that predator exposure induced the activation of CaMKII/CREB, which is accompanied with increased levels of histone acetylation (H3, H4) and decreased histone deacetylases (HDAC1, 2). Subsequently, in the amygdala, the transcription of brain-derived neurotrophic factor (BDNF), neuropeptide Y (NPY) and its Y1 receptor were up-regulated, whereas the Y2 receptor was down-regulated. Therefore, EC facilitated a coping response against a fear associated cue in a PTSD animal model and reduced anxiety by differentially activating serotonergic and NPY-ergic systems.

## Introduction

Stressful life events are potent stressors that promote a fear response. The inability to extinguish memories associated with fear lead to the development of anxiety and post-traumatic stress disorder (PTSD). PTSD is associated with functional changes in the amygdala, a region that mediates fear responses [1–3]. Exposure to predator threats or cues (mouse and rat model/ rat and cat model) has been widely accepted as a model to study PTSD because it relates to innate fear and mimics several behavioral disorders related to PTSD [4–6]. In rodents, earlier studies have demonstrated that reduction in serotonergic transmission influences the development of PTSD directly [7]. Serotonin [5-Hydroxytrypamine, (5-HT)] activity is exerted by Na^+^/ Cl^-^ dependent serotonin transporter (SERT) and 5-HT receptors (5-HT_1_- 5-HT_7_) [8–9].

Additional evidence associates the activation/inactivation of 5-HT receptors with the signaling pathway, in which Ca^2+^-calmodulin-dependent-protein-kinase II (CaMKII) and the transcription factor cyclic adenosine monophosphate (cAMP) response element binding protein (CREB) are known to be involved [10–11]. The activation of the signaling pathway implicated in the chromatin modification [acetylation of histones (H2A, H2B, H3, H4)/ deacetylation] regulates the expression of specific genes [12–13]. Additionally, environmental and social cues also play a role in chromatin modification which in turn initiate the transcription of neuropeptide Y (NPY) and distinct transcripts of brain-derived neurotrophic factor (BDNF) [14–17]. In the amygdala, Y1 and Y2 receptors localize differentially in NPY neurons and thereby critically mediate anxiolytic actions of NPY [18–19].

Interestingly, in animal models it has been shown that enriched condition (EC) improves the development of anxiolytic effects against psychogenic stressors [20–22]. In this study, we hypothesized that EC reduces the predator-threat induced stress. To test our hypothesis, Indian field mouse *Mus booduga* was exposed to their natural predator (field rat *Rattus rattus*) in the rat exposure chamber after housing the individuals in standard condition (SC) or in EC and tested for anxiety. Subsequently, we examined the influence of EC on the serotonergic system (SERT, 5-HT_1A_, 5-HT_2C_) and its pathway-associated signaling molecules, including CaMKII, the transcription factor CREB, molecules involved in chromatin modifications (H3, H4 and HDACs1, 2), the neurotrophin BDNF (exon IV) and the neuropeptide NPY (Y1, Y2 receptor).

## Materials and Methods

### Study Animals

Male field mice *Mus booduga* (Bwt. 7.50 ± 1.44 g; 3 months of age; [23]) and male field rats (*Rattus rattus*; Bwt. 243.66 ± 3.18 g) were trapped using rodent trappers in agricultural land (India, Tiruchirappalli, 10°16’N, 78°15’E). Animals (*M*. *booduga n* = 102 in total; *R*. *rattus n* = 3) were transported to an animal house facility, housed in a standard condition (SC; cage: 43 X 27 X 15 cm) and maintained at controlled environmental conditions (12 h light/dark cycle; 23°C ± 1°C; 50% ± 5% RH) for seven days. Food (Pearl millet *Pennisetum glaucum*) and water were provided *ad libitum* except during brief behavioral test periods. According to the Indian Wildlife Protection Act 1972, *Mus booduga* is classified as vermin and listed in schedule V. Therefore, no specific permission was required to capture them from agricultural land. All the experiments were approved by the Institutional Animal Ethical Committee (BDU/IAEC/2012/73/28.03.2012) in accordance with the guidelines of "Committee for the Purpose of Control and Supervision of Experiments of Animals" of the Government of India. All efforts were made to minimize animal suffering, reduce the number of animals used, and utilize alternatives to *in vivo* techniques, if available.

### Housing Conditions and Experimental Procedures

For the standard conditions, standard laboratory cage (43 X 27 X 15 cm) with saw dust was used as bedding (2 animals/ cage). The cage (120 X 100 X 60 cm) for enriched conditions (EC) was constructed based on the specifications described by Mora et al. [24]. The EC cage (12 animals per cage) contained an assortment of objects varying in shapes, textures and sizes, including running wheels, ladders and hollow plastic pipes with different bends, which were changed twice per week for novelty. To stimulate exploratory activity, the location of toys was changed every day without disturbing the animals, with cleaning once per week. Food and water were kept in a separate polycarbonate cage (43 X 27 X 15 cm) and connected to the EC cage through a transparent plastic pipe (diameter 4.5 cm).

**Fig 1:**
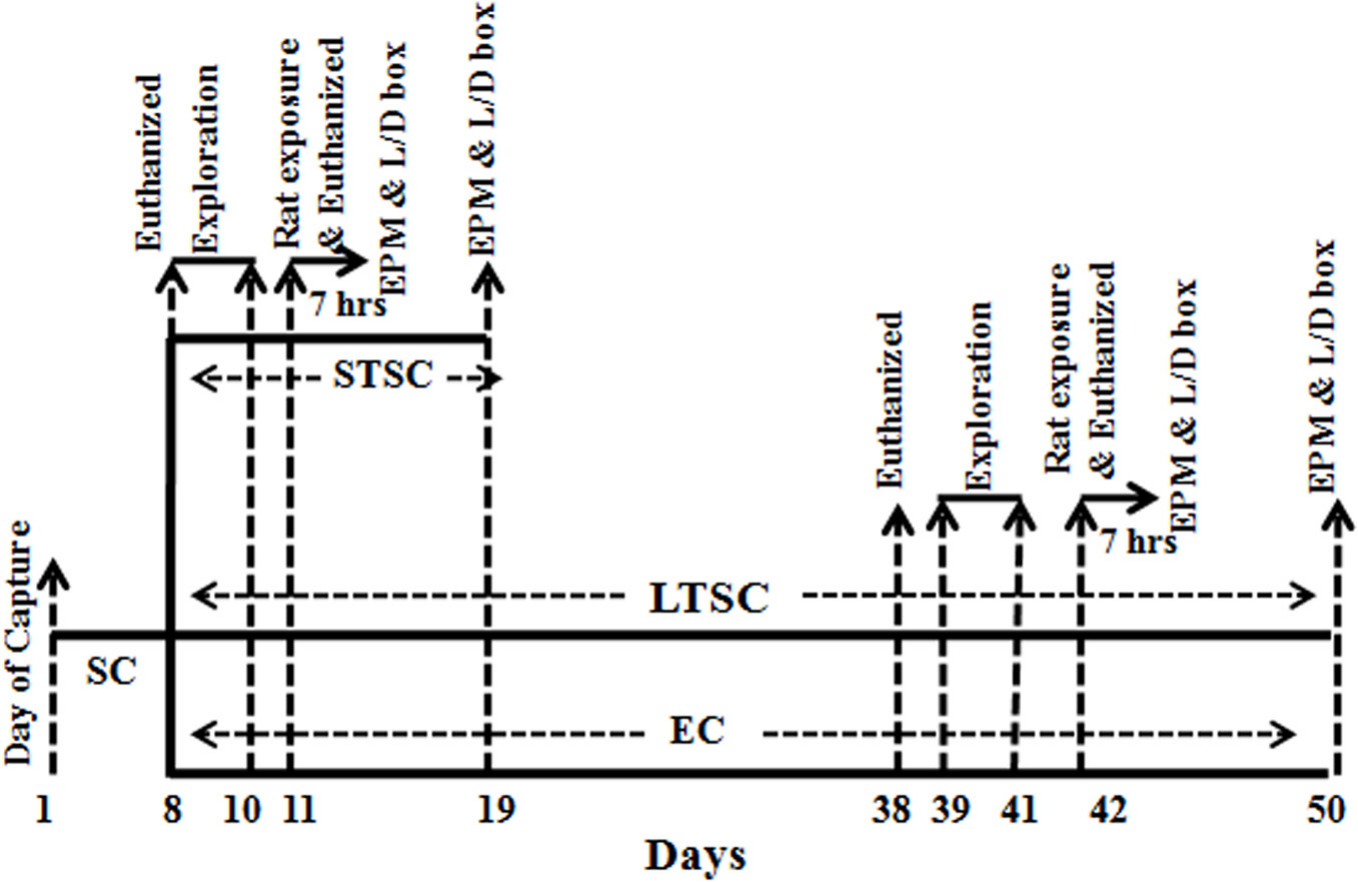
Schematic representation of the experimental procedure used to test the effects of housing condition on predator exposure induced anxiety. After housed in a standard condition (SC; for 7 days), mice were randomly separated to short-term at standard condition (STSC) or long-term at standard condition (LTSC) or enriched condition (EC) and kept in the animal facility until to complete all behavior.

Fig 1 shows the schematic diagram of the experimental procedure. On day 8, animals were randomly divided into three groups (*n* = 34 for each). Group I raised under SC were used as the control group and referred to as short term at standard condition (STSC). One set of animals (*n* = 12) were considered STSC control and immediately euthanized (for neurotransmitter and molecular analysis) without exposure to predators. The second set of animals (*n* = 22) were exposed to the predator (*R*. *rattus*). Their behavior was recorded, and after an hour, one group (*n* = 12) was euthanized (for neurotransmitter and molecular analysis). Remaining animals (*n* = 10) were housed at STSC, and then they were subjected to elevated plus maze (EPM) and light/ dark-box test 7 hrs and 7 days after exposure to test their level of anxiety. Group-II was kept at SC for another 30 days and considered long-term at standard condition (LTSC). Group III animals were transferred to the EC and maintained for another 30 days. On the 31^st^ day, animals from both LTSC (*n* = 12) and EC (*n* = 12) were euthanized without being exposed to the predator, and they were considered as control for LTSC and EC. Subsequently, one set of animals (*n* = 12; for each group) from each housing condition (LTSC/ EC) were exposed to predators individually, and their behavior was recorded. Remaining animals (*n* = 10) from each group were tested for anxiety in EPM and light/ dark-box, 7 hrs and 7 days after exposure to the predator. An hour after being exposed to the predator stimuli, animals were euthanized for neurotransmitter and gene expression analysis.

### Predator Exposure

Field mice (*M*. *booduga*) were exposed to field rat (*R*. *rattus*) in the rat exposure chamber to induce a fear associated anxiogenic effect, and subsequent behavioral and neuronal manifestations are related to PTSD [25]. Briefly, the rat exposure chamber consists of four clear Plexiglas sides covered with a black Plexiglas lid. The exposure chamber was divided by wire mesh into two equal-sized compartments (23 X 12 X 11 cm) as surface area (*i*.*e*., mice exploration area) and predator compartment (*i*.*e*., rat chamber). The exposure chamber was fitted with a home chamber (7 X 7 X 12 cm; three sides with black and one side transparent Plexiglas) through a clear tube (length: 13 cm; diameter: 4.4 cm). The apparatus is a "visible burrow system" model [26] that consists of a home chamber as a place for retreat, a tunnel and a surface area in close contact to the predator exposure chamber (open area).

All the behavioral experiments were conducted at the animal house facility during the light phase (8:00 to 12:00) of the light/ dark cycle in semi-sound proof conditions under red light (100 W). Initially, mice were allowed to explore the surface area for three days (10 min/day) consecutively. On day 4, the mouse was placed in the center of the surface area facing the predator compartment, and then, the rat was released immediately into the predator compartment. The experimental mice were exposed to predators individually for 10 min for one day. When the mouse was exposed to the predator, the mouse entered the home chamber, moved into the tunnel to assess risk by olfactory and visual cues or entered the surface area and then exhibited a range of behaviors (free movement/ defensive). The behavior was recorded using a video camera placed at the horizontal position of the apparatus. The apparatus was cleaned with 70% alcohol and dried with paper towels; respective individual’s cage bedding was poured into the home cage as well as in the surface area for each exposure. The recorded behavior of each mouse was scored as spatiotemporal measures (individual’s time spent in the home chamber, tunnel, on the surface area and in contact with the wire mesh, climbing on the mesh) and ethological measures [freezing (rapid cessation of any body movement during active exploration), stretch attend posture (an exploratory movement in which the body is stretched forward but the animal’s hind paws remains in position), grooming (washing or mouthing of fore limbs, hind paws, face, body and genital) and risk assessment behavior (animal kept its head and two paws facing the predator chamber but the rest of its body in the tunnel)] as described earlier [25,27,28].

### Elevated Plus Maze Test

Elevated-plus-maze (EPM) was constructed with a thin (10 mm) wooden sheet as described earlier [29] [two open arms (30 x 5 x 0.25 cm^3^) and two closed arms (30 x 5 x 15 cm^3^)]. The open and closed arms connected by common central platform (5 x 5 cm^3^) [29]. Animals were transported to the experimental room (luminosity at the level of the EPM was 30 W) an hour before starting the experiment and were left undisturbed. Animals were allowed to explore the maze for 5 min by placing it at the center square facing an open arm. Individual behavior (number of entries into open and closed arms, time spent in open and closed arm, head-dipping, jump attempt and freezing behaviors) was analyzed from the video recordings [30,31]. The animal was transferred to the home cage after the completion of the trail and the maze was cleaned with wet and dry cloths, respectively.

### Light/ dark-box Test

Light/ dark-box was designed based on the previous report [32], and the apparatus (44 x 21 x 21 cm) consisted of a 10 mm wooden sheet box divided into two compartments with a partition of rectangular opening (12 x 5 cm) at the floor level. The larger white painted compartment (28 x 21 cm length) was open-trooped and brightly illuminated (900 lux). The smaller compartment (14 x 21 cm length) was painted black and covered at the top. Animals were transported to the experiment room an hour before starting the experiment and left undisturbed. Animals were allowed to explore the apparatus individually for 5 min by placing the individuals in the light compartment facing away from the partition. The video camera was mounted on the ceiling above the apparatus and facilitated recording of all behaviors (time spent in the light compartment, light-dark transition between compartment and risk assessment) for each individual. To remove any olfactory cues, the apparatus was cleaned with 70% ethanol after the completion of the trial.

### Neurotransmitter analysis

Animals representing each group (*n* = 6) [control (STSC, LTSC and EC), Rat (STSC, LTSC and EC)] were euthanized, and the amygdala region was dissected as described elsewhere [33] and frozen on dry ice. The samples were prepared as reported earlier [34] and the level of 5-HT (Bio-source ELISA Kit) was estimated according to the manufacturer’s instructions.

### Tissue Collection, RNA isolation, and cDNA synthesis

Individuals representing each group (*n* = 6) were euthanized, and the amygdala tissue was dissected for the preparation of total RNA and protein. Total RNA was isolated using TRIzol (Merck Specialties Pvt. Ltd., India), according to the manufacturer’s instructions and stored with RNase inhibitor (GeNei, Merck Specialties Pvt. Ltd., India) at -70°C. Total RNA (2.0 μg/sample) was reverse transcribed into cDNA using random/ oligo-dT primers (iScript cDNA Synthesis Kit, Bio-Rad Laboratories Inc.).

### Quantitative Real-time PCR

Expression levels of specific genes were quantified by quantitative real-time PCR (qRT-PCR) in a total volume of 10 μl containing an aliquot of RT mixture (SSoAdvanced SYBR green SuperMix, Bio-Rad Laboratories Inc.), specific primers (10 μM) and cDNA (0.1 μg). The qRT-PCR reactions were performed using CFX-96 Touch Real-time PCR Detection System (Bio-Rad Laboratories Inc.) using standard conditions. The following specific primers were used for each gene: 5-HT_1A_ (for 5’-GACTACGTGAACAAGAGGAC-3’ and rev primer 5’-TATAGAAAGCGCCGAAAGTG-3’), 5-HT_2C_ (for 5’-AAACTGCAC AATGCTACCAA-3’ and rev primer 5’-TGATGGACGCAGTTGAAAAT -3’), NPY (for 5’-GGACATGGCCAGATACTACT-3’ and rev primer 5’-CTTCAAGCCTTGTTCTGGG-3’), NPY Y1 (for 5’-TGAACCTCTCCTTCTCAGAC-3’ and rev primer 5’-TTGATGATTAGC TGATGCCG-3’), NPY Y2 (for 5’-ATCGTTGCATTGTCTACCAC-3’ and rev primer 5’- TACACACTCTTCTCTTCCCC-3’), BDNF exon IV (for 5’-GTAAACGTCCACGGACAA G-3’ and rev primer 5’-TATGGTTTTCTTCGTTGGGC-3’) and GAPDH (glyceraldehydes-3-phosphate dehydrogenase) (for 5’-AACATCATCCCTGCATCCAC-3’ and rev primer 5’-AGGAACACGGAAGGCCATGC-3’). The amplification of the single PCR product was confirmed by monitoring the dissociation curve followed by melting curve analysis. Each reaction was performed in triplicate with threefold serial dilution of cDNA with normalizing internal control GAPDH. The data are presented as the mean fold change of the relative expression (CFX Manager version 2 software; CFX-96 Touch Real-time PCR Detection System Bio-Rad Laboratories Inc., USA).

### Protein Isolation and Western Blot Analysis

Total cellular protein extract from different samples were prepared following the standard procedures and stored as aliquots at -80°C [35]. The histone proteins were extracted from amygdala tissue following the method described previously [36]. The concentration of each protein sample was quantified by measuring the absorbance at 595 nm using Biophotometer plus (Eppendorf Inc). Equal concentrations (60 μg) of proteins were resolved on SDS-polyacrylamide gel. The separated proteins were transferred electrophoretically onto polyvinylidene difluoride (PVDF) membrane (Millipore India) using a semi-dry western apparatus (SD 20; Cleaver Scientific Ltd, UK). The membranes were blocked and incubated at 4°C for 9 h with one of the following specific primary antibodies (Santa-Cruz Biotechnology, Germany and Upstate, Cell signaling solutions, USA): affinity purified rabbit polyclonal antibody anti-total-CREB1 [t-CREB 1 (SC-186; 1: 300], rabbit polyclonal anti-phosphorylated-CREB1 antibody [p-CREB 1; Ser^133^(SC-101663; 1: 500)], mouse monoclonal anti-total-αCaMKII antibody [t-αCaMKII SC-32288; 1: 200], rabbit polyclonal anti-phosphorylated- αCaMKII antibody [p-αCaMKII; Thr^286^ SC-12886-R; 1: 200], goat polyclonal antibody SERT [ST; SC-1458; 1: 200], rabbit polyclonal acetylated histone H3 antibody [Ac-Histone H3, (Cell Signaling—# 06–599; 1:5000)], rabbit polyclonal acetylated histone H4 antibody [Ac-Histone H4 (Ser1/Lys5/Lys8/Lys12), (SC-34263; 1:400)], mouse monoclonal Anti-HDAC-1 antibody [Anti-HDAC-1, clone 2E10, (Cell Signaling # 05–614; 1:5000)], mouse monoclonal Anti-HDAC-2 antibody [Anti-HDAC-2, clone 3F3, (Cell Signaling # 05–814; 1:5000)] and rabbit polyclonal anti-β-actin antibody (SC-130656; 1: 200) as internal control for each sample. The membrane-bound antibodies were detected by alkaline phosphatase (ALP) conjugated either with goat anti-rabbit (Cat # 621100180011730; 1: 2000; MERCK, Bangalore, India), goat anti-mouse (Cat # 621100480011730; 1:2000; MERCK, Bangalore, India) or rabbit anti-goat (Cat # 621100680011730; 1:2000; MERCK, Bangalore, India) secondary antibody incubating for 4 h. Images were acquired with a Molecular Imager ChemiDoc XRS System (Bio-Rad Laboratories, Inc, USA), and optical density of trace quantity for each band was measured using Image Lab 2 software (Bio-Rad Laboratories, Inc.).

### Statistical Analysis

Data were presented as a mean ± standard error of the mean (SEM) and plotted with KyPlot (ver 1.0) for graphical representation. For fear response One-way analysis of variance (ANOVA) was used to test the effect of housing condition. For expression data Two-way analysis of variance (Two-way ANOVA) was used to assess the effect of ‘exposure’ and ‘housing condition’ and their interactions. For the behavioural analysis (EPM, Light/dark-box) multivariate ANOVA was performed to test the effect of factors (exposure, time, housing condition) and their interactions. *Post hoc* Bonferroni test was used to examine the difference between groups (SPSS, ver.15).

## Results

### Effect of environmental enrichment to a predator exposure

#### Fear response behavior

**Fig 2:**
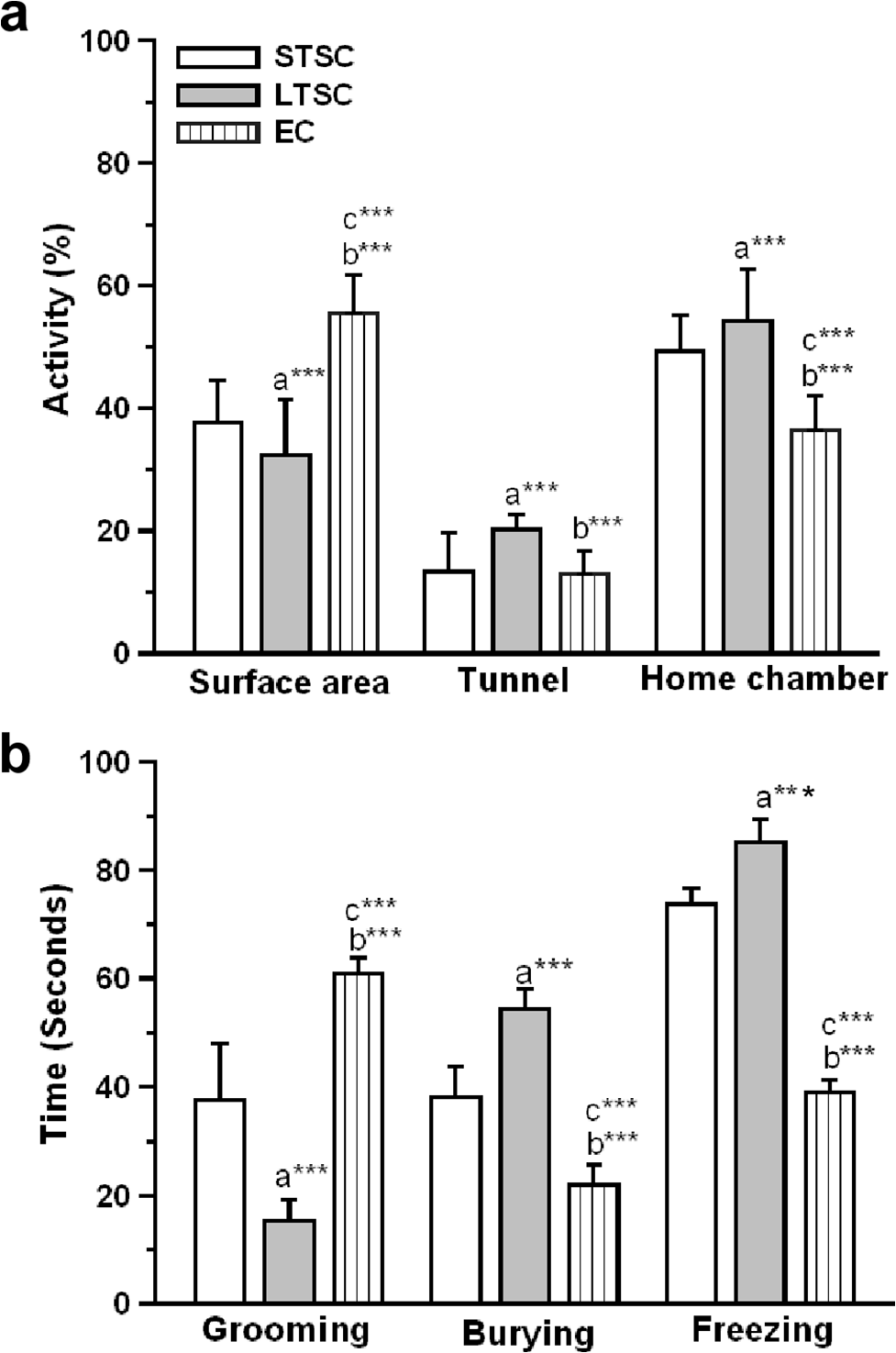
Effect of environmental enrichment on the behavioral responses of mice that were exposed to the predator. The exposure chamber is an area for mice to explore and investigate about the predator but the mesh partition prevents interactions. The behavioral activities of mice during exposure to a rat were shown. The columns in the graph represent duration of mice activity in respective region during predator exposure session. Data were shown as mean ± SEM, * indicates significant difference (*P < 0.05; **P < 0.01; ***P < 0.001). The letters indicates groups were compared for statistical analysis (a: STSC verses LTSC; b LTSC verses EC; c: STSC verses EC). Raw data is provided in S1 Data.

Mice housed under various environmental conditions were exposed to a predator in rat exposure chamber. The location and time spent in a specific area of the test chamber in response to a predator was determined. Spatiotemporal measures showed that predator exposure rapidly elicited innate fear response in STSC and LTSC mice but not in EC mice (Fig 2). The One-way ANOVA analysis revealed a significant effect of housing condition in exploratory behaviour on surface area (F_2, 65_ = 521.21, *P*<0.001). Further, the Bonferroni test confirmed that EC mice spent a significantly longer duration on surface area compared with STSC (*P<*0.001) and LTSC mice (*P*<0.001). As shown in Fig 2A, STSC mice showed more exploratory behavior on surface area and spent more time in surface area than LTSC mice (*P*<0.001). Similarly, we found significant effect of housing condition in individuals spent time in tunnel (F_2, 65_ = 52.20, *P*<0.001). In comparison, EC mice spent significantly less time than LTSC (*P*<0.001), but the difference was not significant in STSC mice (*P*<0.084). Interestingly, STSC mice spent significantly less time in the tunnel than LTSC mice (*P*<0.01). The housing condition had significant effect on time spent by mice in the home chamber (F_2, 65_ = 807.23, *P*<0.001). Furthermore, the EC mice preferred to spend significantly less time in the home chamber than STSC (P<0.001) and LTSC mice (*P*<0.001). Compared with STSC mice, time spent by STSC mice in the home chamber was significantly less than LTSC mice (*P*<0.001). Additionally, we observed effect of housing condition on grooming behavior (F_2, 65_ = 133.66, *P*<0.001), burying (F_2, 65_ = 167.18, *P*<0.001) and freezing (F_2, 65_ = 633.08, *P*<0.001). EC mice displayed significantly more grooming behavior than the STSC (*P*<0.001) and LTSC (*P*<0.001) mice. Interestingly, EC mice showed less reactive behaviors, such as defensive behavior (burying) and freezing than STSC (burying: *P*<0.001; freezing: *P*<0.001) and LTSC mice (burying: *P*<0.001; freezing: *P*<0.001). However, STSC mice showed less reactive behaviors than LTSC mice (burying: *P*<0.001; freezing: *P*<0.001). Taken together, the EC and STSC mice displayed less fear response during the predator exposure than LTSC mice as indicated by staying more in surface area by exploring the exposure chamber than the tunnel/ home chamber.

### Anxiety-like behavior

#### Elevated plus maze (EPM)

Multivariate ANOVA analysis showed that the mice exhibited behaviors 7hrs and 7 days (time) after rat exposure was significantly different (Wilks’ λ = 0.447; F = 4.95; *P*<0.01). Similarly, the housing condition (Wilks’ λ = 0.011; F = 33.76; *P*<0.001) and interaction of time × housing condition (Wilks’ λ = 0.091; F = 9.28; *P*<0.001) showed significant effects on behavior. However, the independent analysis showed that time influence only few behaviors but not all. Whereas the interaction of time × housing condition effect was significant in all observed behavior except the time spent in central square (Table 1).

**Table 1:** Elevated plus-maze test profile of male field mouse *Mus booduga*.

Parameters	Housing Conditions	Time		Statistics (F value)			Bonferroni test
		7hrs	7days	Between Time	Between Groups	Interaction: Time X Housing Conditions	
No. of open arm entries	STSC	14.00 ± 1.62	8.30 ± 1.21	0.426^NS^	56.923^***^	8.283^**^	a: *P* = 0.139
	LTSC	7.60 ± 0.96	7.30 ± 1.67				b: *P*<0.001
	EC	21.30 ± 2.45	30.20 ± 2.42				c: *P*<0.001
Time spent in open arm (s)	STSC	156.90 ± 5.73	81.80 ± 4.78	20.802^***^	256.334^***^	60.928^***^	a: *P*<0.001
	LTSC	76.90 ± 3.78	80.20 ± 5.69				b: *P*<0.001
	EC	172.00 ± 4.46	192.30 ± 2.27				c: *P*<0.001
Freezing in open arm (s)	STSC	29.50 ± 2.43	52.30 ± 4.62	3.850^*^	79.874^***^	11.112^***^	a: *P*<0.01
	LTSC	52.10 ± 4.13	51.70 ± 4.26				b: *P*<0.001
	EC	15.00 ± 0.94	8.40 ± 1.19				c: *P*<0.001
No. of closed arm entries	STSC	15.50 ± 1.76	19.90 ± 1.58	0.568^NS^	41.676^***^	5.596^*^	a: *P*<0.01
	LTSC	25.10 ± 1.72	19.40 ± 1.44				b: *P*<0.001
	EC	9.40 ± 1.24	7.90 ± 1.13				c: *P*<0.001
Time spent in closed arm (s)	STSC	100.30 ± 3.45	166.20 ± 6.13	13.885^***^	174.571^***^	41.768^***^	a: *P*<0.001
	LTSC	169.90 ± 6.89	168.80 ± 5.72				b: *P*<0.001
	EC	87.80 ± 2.43	68.00 ± 3.17				c: *P*<0.001
Freezing in closed arm (s)	STSC	20.40 ± 2.82	31.20 ± 2.08	0.748^NS^	50.916^***^	8.596^**^	a: *P*<0.01
	LTSC	34.00 ± 2.78	35.50 ± 2.51				b: *P*<0.001
	EC	16.30 ± 1.27	8.70 ± 1.25				c: *P*<0.001
Head-dipping in closed arm (s)	STSC	57.60 ± 3.67	82.20 ± 1.84	13.562^**^	141.185^***^	5.653^*^	a: *P*<0.001
	LTSC	80.40 ± 3.95	81.20 ± 1.92				b: *P*<0.001
	EC	29.10 ± 2.44	19.70 ± 2.60				c: *P*<0.001
Time spent in central square (s)	STSC	42.60 ± 5.60	52.30 ± 8.04	0.179^NS^	1.652^NS^	0.455^NS^	a: *P* = 1.000
	LTSC	53.20 ± 7.56	51.00 ± 9.01				b: *P* = 0.232
	EC	40.20 ± 4.10	39.70 ± 4.68				c: *P* = 0.813
No. of jump attempts	STSC	10.70 ± 0.94	2.50 ± 0.50	1.171^NS^	91.067^***^	16.602^***^	a: *P*<0.01
	LTSC	2.50 ± 0.92	1.80 ± 0.59				b: *P*<0.001
	EC	15.10 ± 1.40	20.80 ± 2.10				c: *P*<0.001

Data are presented as means ± SEM *n* = 10 mice/group (STSC—short-term standard cage, LTSC—long term-standard cage, EC—enriched condition). Data were subjected to MANOVA for multiple comparisons.

(*** *P*<0.001

** *P*<0.01

* *P*<0.05

NS, not significant).

Bonferroni test (a: STSC vs LTSC, b: LTSC vs EC, c: STSC vs EC). Raw data is provided in S1 Data.

#### Light/ dark-box test

**Fig 3:**
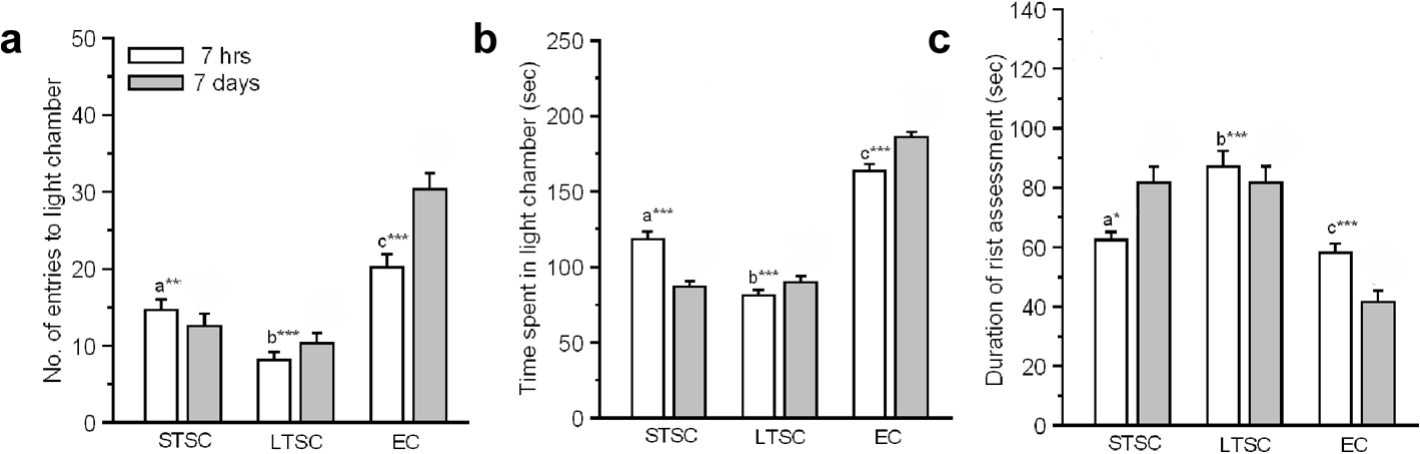
Behavioural profile of mice in light/dark-box test after housed in environmental enrichment exposed to predator. (a) EC made more entries to light chamber after seven hours and seven days of predator exposure and exert anxiolytic effect compared to STSC and LTSC mice. (b) EC mice spent more time in light zone compared to STSC and LTSC mice. (c) EC mice exhibit few risk assessment behaviour than other groups of mice. Data are presented as means ± SEM, * indicates significant difference (***P < 0.001; **P < 0.01; *P < 0.05), respect to comparison between groups a = STSC versus LTSC: b = LTSC verses EC: c = STSC verses EC. Raw data is provided in S1 Data.

The effect of predator exposure on anxiety in individuals housed in various conditions was tested using a light/ dark-box test. Multivariate ANOVA analysis revealed that individuals behavior significantly affected by time (Wilks’ λ = 0.789; F = 4.64; *P*<0.01), housing condition (Wilks’ λ = 0.07; F = 46.03; *P*<0.001) and interaction of time × housing condition (Wilks’ λ = 0.41; F = 9.70; *P*<0.001) (Fig 3). Furthermore, our analysis revealed significant effect of time (F_1,59_ = 7.69; *P*<0.01), housing condition (F_2,59_ = 58.54; *P*<0.001) and interaction of time × housing condition (F_2,59_ = 8.20; *P*<0.01) in mice made entries to the light chamber. Follow-up post hoc Bonferroni test confirmed that EC mice made more entries to the light chamber than STSC (*P*<0.001) and LTSC mice (*P*<0.001). In comparison, STSC made more entries into the light zone than LTSC mice (*P* = 0.021). Our analysis revealed that the time spent in the light zone did not affected by time (F_1,59_ = 0.001; *P* = 0.97). However, we found significant effect by housing condition (F_2,59_ = 268.47; *P*<0.001) and interaction of time × housing condition (F_2,59_ = 23.67; *P*<0.001). Bonferroni test showed that EC mice spent more time in the light zone compared with STSC (*P*<0.001) and LTSC mice (*P* < 0.001). In addition, STSC mice spent more time in the light zone compared with LTSC (*P*<0.001). Interestingly, the time of testing (F_1,59_ = 7.87; *P*<0.01), housing condition (F_2,59_ = 31.18; *P*<0.001) and interaction of time × housing condition (F_2,59_ = 4.60; *P*<0.05) effectively alter the risk assessment behavior. Bonferroni test confirmed that EC mice spent significantly less time in risk assessment than STSC (*P*<0.001) and LTSC mice (*P*<0.001). However, STSC mice displayed less risk assessment behavior than LTSC mice (*P* < 0.05).

### Effect of environmental enrichment on the activation of serotonergic system in mice exposed to a predator

**Fig 4:**
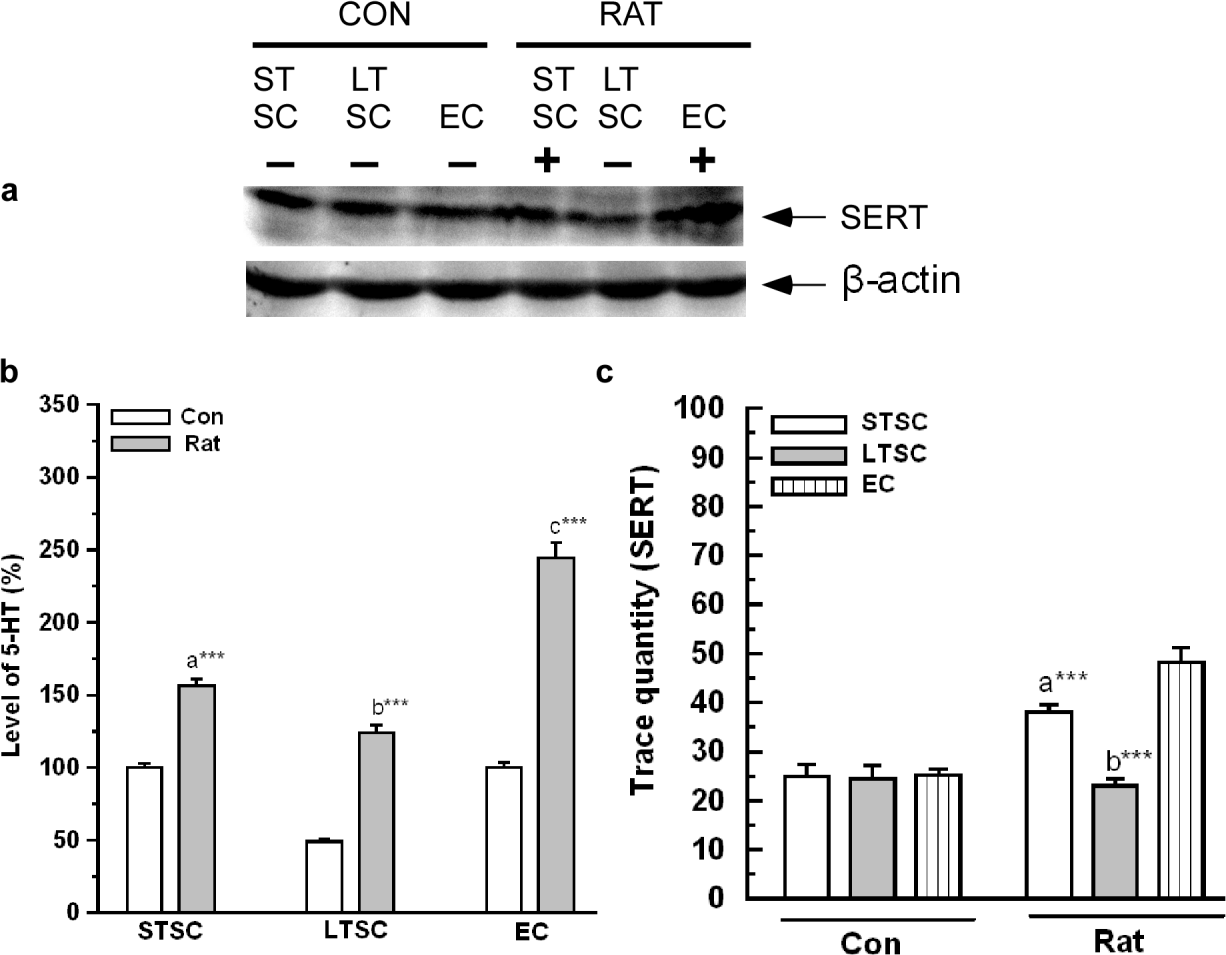
Effect of environmental enrichment on activation of SERT and 5-HT level in mice exposed to predator. (a) Representative western blots shows the level of SERT (70 kDa) and β-actin (43 kDa). (b) There was a significant increase in 5-HT level after exposure to predator in STSC, LTSC and EC mice. (C) SERT protein level was significantly increased in EC following predator exposure than STSC and LTSC mice. Data were shown as mean ± SEM, * indicates significant difference (*P < 0.05; **P < 0.01; ***P < 0.001), respect to comparison between groups (a = STSC verses LTSC; b = LTSC verses EC; c = STSC verses EC). Raw data is provided in S2 Data.

We determined the level of 5-HT, expression level of SERT, 5-HT_1A_ and 5-HT_2C_ in the mice housed in various environmental conditions and exposed to a predator. As shown in Fig 4A, basal levels of 5-HT were significantly affected by exposure (F_1,35_ = 1811.16; *P*<0.001), housing condition (F_2,35_ = 431.95; *P*<0.001) and interaction of exposure × housing condition (F_2,35_ = 147.85; *P*<0.001). Further, Bonferroni test revealed that the 5-HT level was significantly higher in EC mice (*P*<0.001) and STSC mice (*P*<0.001) than LTSC mice. In comparison, elevated levels of 5-HT were significantly higher in STSC mice than LTSC mice (*P*<0.001).

Interestingly, SERT level was significantly up-regulated after rat exposure in all the mice irrespective of their housing condition. The analysis revealed a significant effect of exposure (F_1,35_ = 35.60; *P*<0.001), housing condition (F_2,35_ = 18.89; *P*<0.001) and interaction of exposure × housing condition (F_2,35_ = 16.17; *P*<0.001) in the level of SERT (Fig 4A and 4C). Further, Bonferroni test showed that level of SERT significantly increased in EC mice compared with LTSC (*P* <0.001), but not different from STSC mice (*P* = 0.296). However, the difference between LTSC and STSC was significantly different (*P*<0.001).

**Fig 5:**
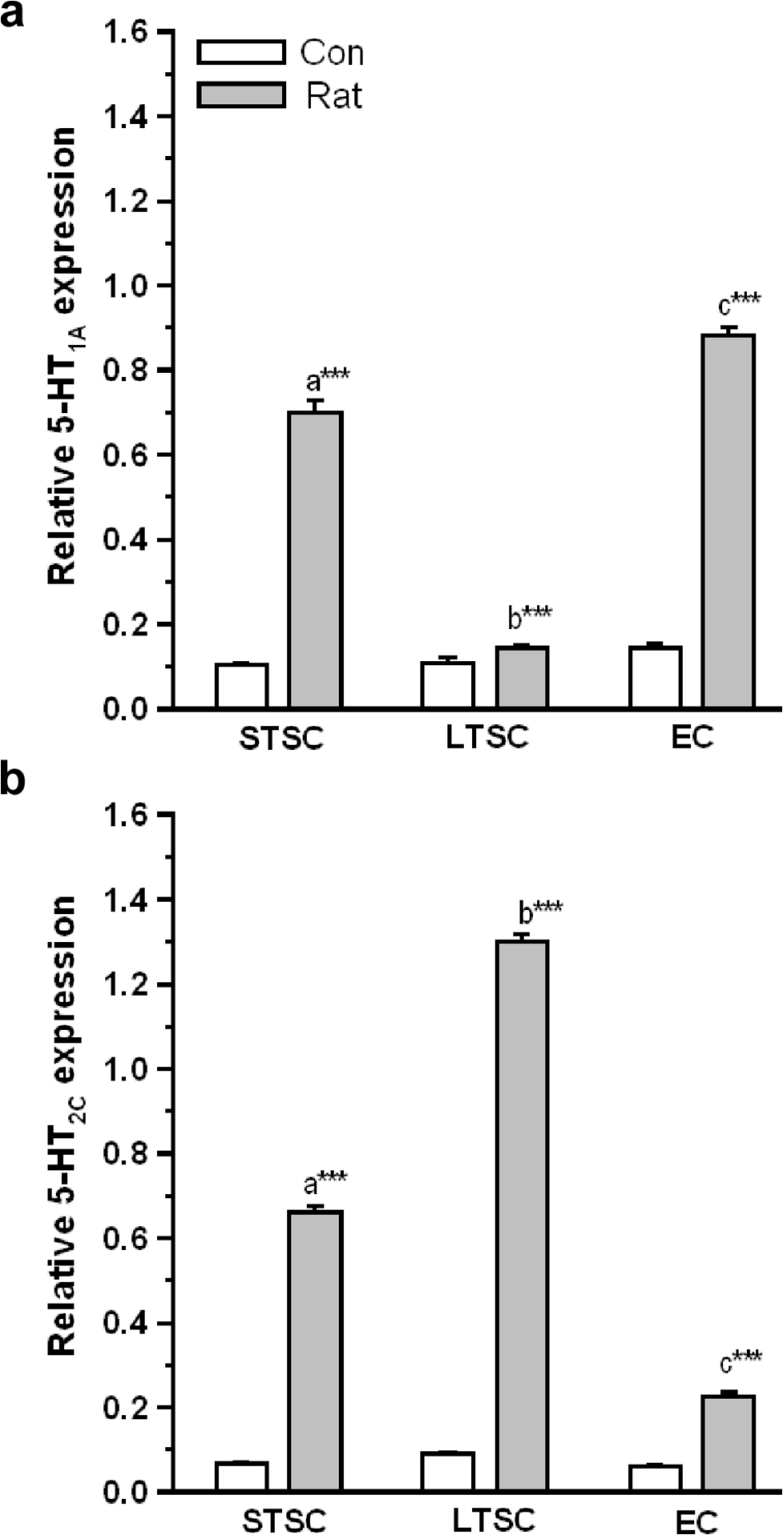
Effect of environmental enrichment on activation of 5-HT1A and 5-HT2C receptors in mice exposed to predator. (a) 5-HT1A receptor mRNA level significantly increased in STSC and EC mice, an effect were not noticed in LTSC mice. (b) 5-HT2C receptor mRNA level was significantly altered in STSC, EC mice but it was significantly high in LTSC mice. Data were shown as mean ± SEM, * indicates significant difference (*P < 0.05; **P < 0.01; ***P < 0.001), respect to comparison between groups (a = STSC verses LTSC; b = LTSC verses EC; c = STSC verses EC). Raw data is provided in S2 Data.

Quantitative RT-PCR analysis was used to examine the variations in the expression of 5-HT receptor mRNA levels in the amygdala (Fig 5). The level of 5-HT_1A_ mRNA level was significantly affected by exposure (F_1,35_ = 872.30; *P*<0.001), housing condition (F_2,35_ = 219.74; *P*<0.001) and interaction of exposure × housing condition (F_2,35_ = 204.17; *P*<0.001). Predator exposure up-regulated 5-HT_1A_ mRNA level in STSC mice and EC mice compared with their respective controls, but did not alter the level of 5-HT_1A_ expression in LTSC mice (Fig 5A). Further Bonferroni test confirmed that the estimated level was significantly higher in EC mice (*P* < 0.001) and STSC mice (*P*<0.001) than LTSC mice. However, the up-regulated 5-HT_1A_ mRNA level was significantly higher in EC mice (*P*<0.001) than STSC mice.

Similar to that of 5-HT_1A_ expression, the level of 5-HT_2C_ expression was significantly affected by exposure (F_1,35_ = 322.18; *P*<0.001), housing condition (F_2,35_ = 78.50; *P*<0.001) and interaction of exposure × housing condition (F_2,35_ = 74.19; *P*<0.001). Significant difference between the group was confirmed by Bonferroni test, we found that the level of 5-HT_2C_ expression was significantly lower in EC mice compared with LTSC mice (*P* < 0.001) and STSC mice (F_1,11_ = 1056.25, *P*<0.001). Interestingly, 5-HT_2C_ receptor expression was significantly low in STSC mice (*P* < 0.001) compared with LTSC mice (Fig 5B).

Our analysis suggests that predator exposure increased extracellular levels of 5-HT and SERT and 5HT_1A_ transcription rapidly in the amygdala of mice housed in the STSC and EC conditions. In contrast, 5-HT_2C_ transcript level was reduced in EC mice, which suggests that housing condition influenced 5-HT signaling in the amygdala.

### Effect of environmental enrichment on the activation of CaMKII/ CREB in mice exposed to a predator

**Fig 6:**
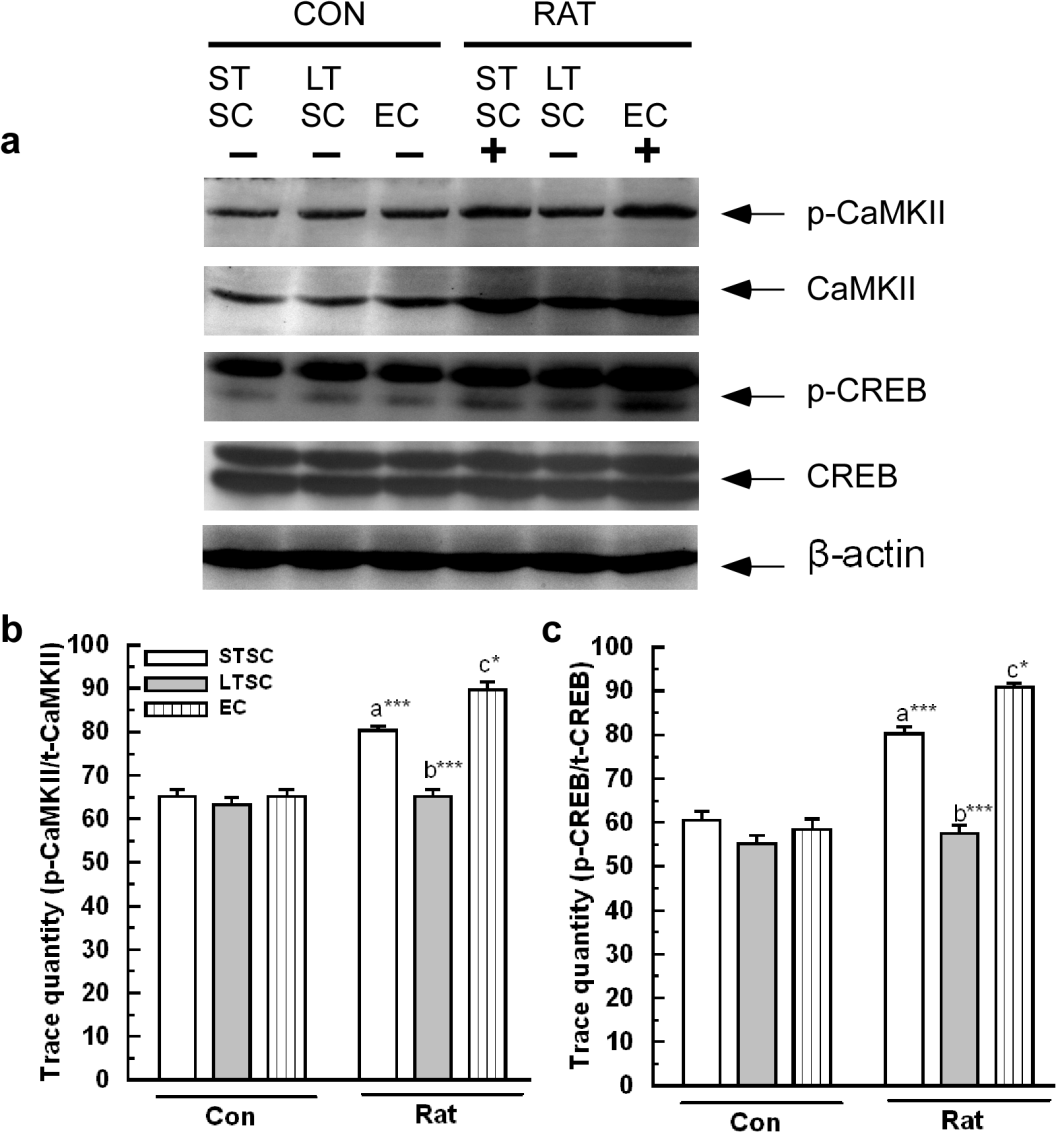
Effect environmental enrichment on activation of CaMKII/CREB in mice exposed to predator. (a) Representative western blots showing the activation (measured as phosphorylated level) of CaMKII (50 kDa) and CREB (43 kDa) after exposed to predator. Phosporylation of (b) CaMKII, (**c**) CREB increased after predator exposure in STSC, LTSC and EC mice The fold variation shown as mean ± SEM. * Indicates significant difference (*P < 0.05; **P < 0.01; ***P < 0.001) respect to comparison between groups (a = STSC verses LTSC; b = LTSC verses EC; c = STSC verses EC). Raw data is provided in S2 Data.

As previously mentioned, the activation of CaMKII/ CREB signaling is associated with 5-HT transmission [11] and PTSD [37]. We examined the variation in the level by phosphorylation level of CaMKII/ CREB (Fig 6A). The relative ratios of p-CaMKII/ CaMKII reflected the level of activation (Fig 6B). We found that the exposure showed significant effect on phosphorylation of CaMKII (F_1,35_ = 107.97; *P*<0.001) and housing condition (F_2,35_ = 33.53; *P*<0.001). In addition, significant effect was observed in exposure × housing condition interaction (F_2,35_ = 24.41; *P*<0.001). Bonferroni test confirmed that the estimated relative p-CaMKII level was significantly higher in predator exposed group housed in EC mice than LTSC mice (*P*<0.001) and STSC mice (*P*<0.05). Moreover, the predator stimuli induced p-CaMKII level was significantly higher in STSC mice (*P*<0.001) than LTSC mice. Accordingly, the exposure to a predator (F_1,35_ = 150.40; *P*<0.001) and housing condition (F_2,35_ = 55.35; *P*<0.001) significantly enhanced the level of p-CREB (Fig 6C). Further, our analysis revealed significant effect by interaction of exposure × housing condition (F_2,35_ = 34.18; *P*<0.001). When the comparisons were made using Bonferroni test between groups, we found that p-CREB level was significantly higher in EC mice (F_1,11_ = *P*<0.001) than LTSC mice but not different from STSC mice (*P* = 0.09). However, predator stimuli induced enhancement of p-CREB was significantly higher in STSC mice (*P*<0.001) compared with LTSC mice. Observed the activation of CaMKII and CREB suggests that long term stay in the standard housing condition moderately suppressed the activation of CaMKII/ CREB in STSC mice.

### Effect of environmental enrichment on chromatin modification in mice exposed to a predator

**Fig 7:**
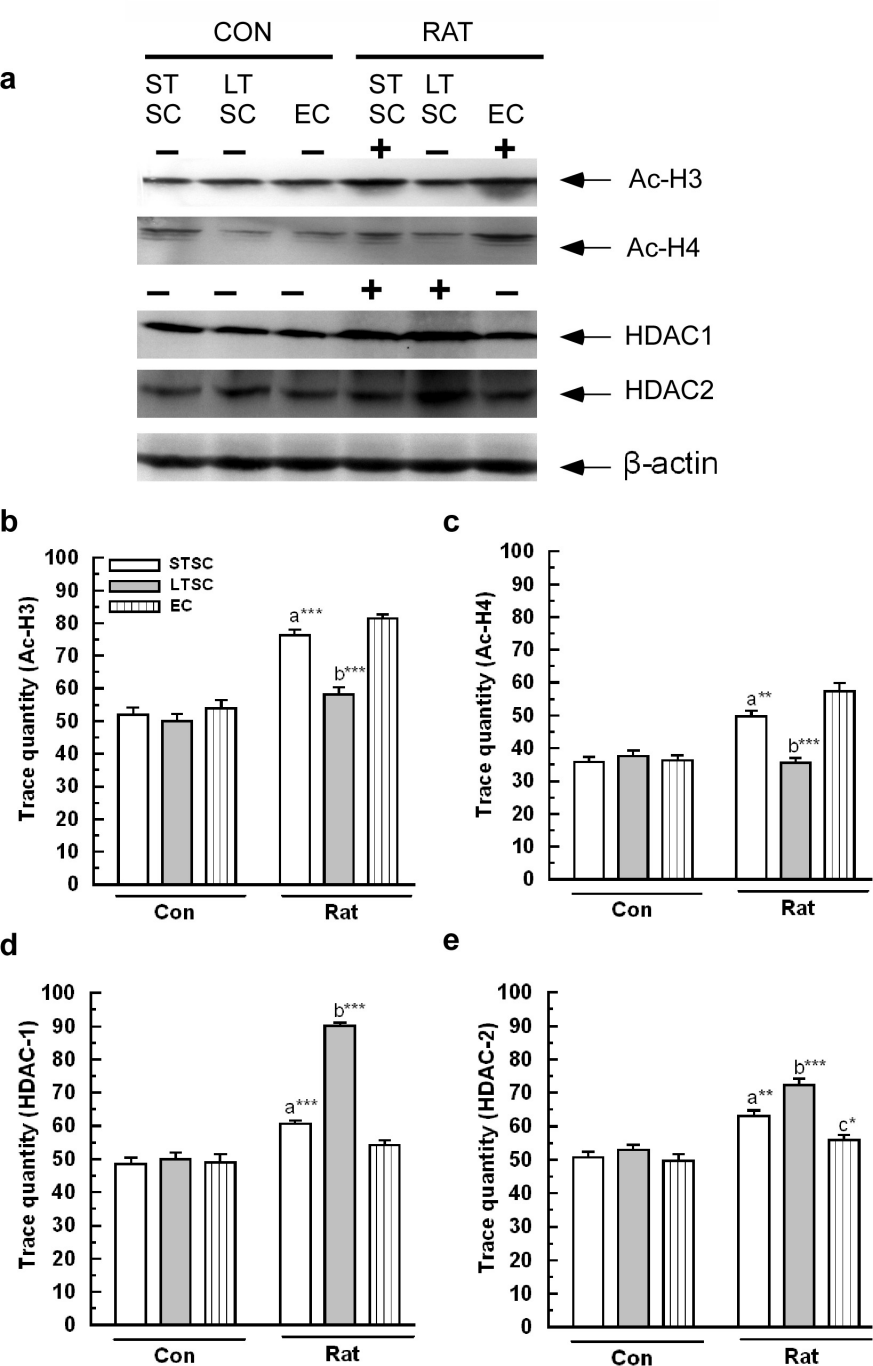
Effect of environmental enrichment on chromatin modifications (alternation in histone acetylation and deacetylation) in mice exposed to predator. (a) Representative western blots shows the levels of H3 (17 kDa), H4 (11 kDa), HDAC-1(60 kDa) and HDAC-2(60 kDa) and β-actin (43 kDa). (b) H3 (**c**) H4 acetylation was significantly increased in STSC and EC mice but not in LTSC mice. While, histone deactylase (d) HDAC1 (**e**) HDAC2 significantly altered in STSC and LTSC mice but not in EC mice. The fold variation shown as mean ± SEM. * Indicates significant difference (*P < 0.05; **P < 0.01; ***P < 0.001) respect to comparison between groups (a = STSC verses LTSC; b = LTSC verses EC; c = STSC verses EC). Raw data is provided in S2 Data.

We tested whether the housing condition altered the histone acetylation/ deacetylation. We found that housing condition alone had no effect on either the acetylation of H3/ H4 or level of HDAC1 and HDAC2 (Fig 7A). Our analysis revealed that acetylation of H3 significantly affected by exposure (F_1,35_ = 131.64; *P*<0.001), housing condition (F_2,35_ = 19.78; *P*<0.001) and interaction of exposure × housing condition (F_2,35_ = 15.58; *P*<0.001). Furthermore, Bonferrroni test confirmed that acetylated H3 level was significantly higher in EC mice (*P*<0.001) and STSC mice (*P*<0.001) than LTSC mice. There were no significant difference between EC and STSC mice (*P* = 0.265) (Fig 7B). Similarly, exposure (F_1,35_ = 61.42; *P*<0.001), housing condition (F_2,35_ = 17.65; *P*<0.001) and interaction of exposure × housing condition (F_2,35_ = 23.09; *P*<0.001) led to significant effect on level of H4 acetylation. In comparing groups, post hoc Bonferroni test confirmed that acetylated H4 level was significantly higher in EC mice (*P*<0.001) and STSC mice (*P*<0.01) than LTSC mice (Fig 7C). However, there were no significant difference in the acetylated H4 level (*P* = 0.079) was not significantly higher in EC mice than STSC mice.

Furthermore, our analysis (Fig 7A) showed variation in the HDAC1 level. We found significant effect of exposure (F_1,35_ = 188.50; *P*<0.001), housing condition (F_2,35_ = 56.59; *P*<0.001) and interaction of exposure × housing condition (F_2,35_ = 60.82; *P*<0.001) in the HDAC1 level. Bonferroni test confirmed that after exposure to the predator the estimated relative HDAC1 level was significantly higher in LTSC mice (*P*<0.001) than EC mice and STSC mice (*P*<0.001), but there were no significant difference between EC and STSC mice (*P* = 0.273) (Fig 7D). Similarly, HDAC2 level was also significantly altered by exposure (F_1,35_ = 90.36; *P*<0.001), housing condition (F_2,35_ = 18.34; *P*<0.001) and interaction of exposure × housing condition (F_2,35_ = 8.3; *P*<0.01). Interestingly, Bonferroni test confirmed that the estimated HDAC2 level was significantly higher in LTSC mice than EC mice (*P*<0.001) and STSC mice (*P*<0.01) (Fig 7E). We found that HDAC2 level was significantly low in EC mice (*P*<0.05) compared with STSC mice. Our results suggest that EC enhances the histone acetyltransferase activity, which enhances the acetylation of H3 and H4 levels. On the other hand, EC possibly inhibited HDAC1 and 2 activities than the other housing condition.

### Effect of environmental enrichment on the activation of neuropeptide Y (NPY)-ergic in mice exposed to a predator

**Fig 8:**
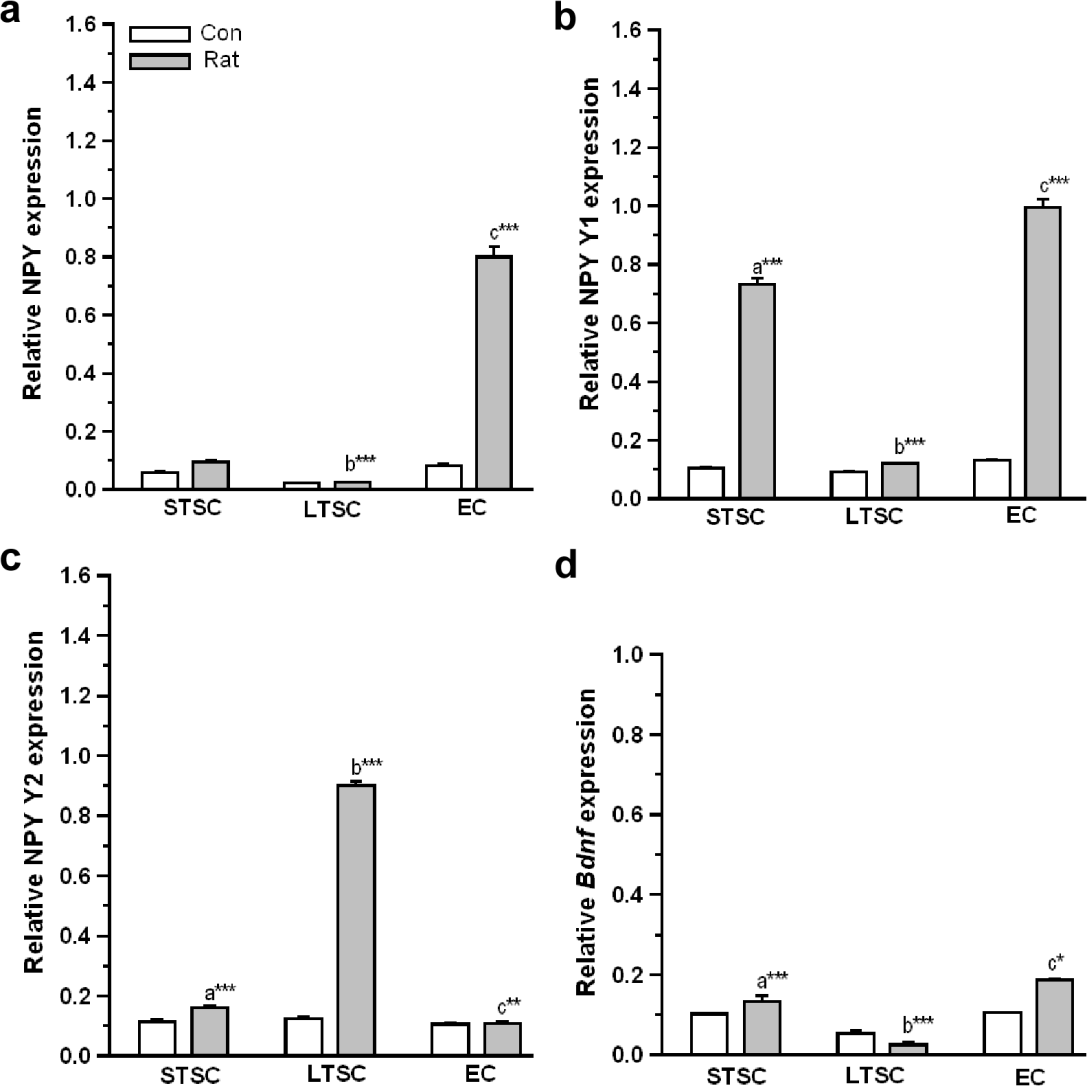
Effect of environmental enrichment on anxiolytic/anxiety related markers in neuropeptide—Y (NPY) ergic system and BDNF in mice exposed to predator. Real-time qPCR showing increase in (a) NPY and (b) NPY Y1 receptor mRNA level in STSC, EC mice but not in LTSC mice. Whereas, NPY Y2 receptor (c) mRNA increased only in LTSC mice but not in STSC and EC mice. (e) BDNF (exon IV) mRNA level increased in STSC and EC mice but not in LTSC mice. The fold variation shown as mean ± SEM. * Indicates significant difference (*P < 0.05; **P < 0.01; ***P < 0.001) respect to comparison between groups (a = STSC verses LTSC; b = LTSC verses EC; c = STSC verses EC). Raw data is provided in S2 Data.

To determine whether the housing and predator exposure alters the neuropeptide transcript expression, we measured the NPY mRNA using quantitative real time PCR (Fig 8A). The analysis showed that there was a significant effect in the expression of NPY in exposure (F_1,35_ = 64.03; *P*<0.001), housing condition (F_2,35_ = 65.61; *P*<0.001) and an exposure × housing condition interaction (F_2,35_ = 62.62; *P*<0.001). When the groups were compared, Bonferroni test revealed that up-regulated NPY mRNA level was higher in EC mice (*P*<0.001) and STSC mice (*P*<0.001) than LTSC mice. However, the estimated up-regulation was not significantly higher in STSC mice (*P* = 0.207) than LTSC mice.

Subsequently, differential expression pattern of the Y1 receptor (Fig 8B) was observed. The expression of Y1 receptor was significantly affected by exposure (F_1,35_ = 3301.03; *P*<0.001), housing condition (F_2,35_ = 907.64; *P*<0.001) and interaction of exposure × housing condition (F_2,35_ = 811.06; *P*<0.001). Further, the Bonferroni test confirmed the difference between groups. The analysis showed that up-regulated Y1 receptor mRNA level was significantly higher in EC mice than STSC mice (*P*<0.001) and LTSC mice (*P*<0.001). Notably, Y1 receptor expression was significantly higher in STSC than LTSC mice (*P*<0.001).

As shown in Fig 8C, difference was found in Y2 receptor expression between the groups representing different housing conditions. We found that Y2 receptor expression was significantly affected by exposure (F_1,35_ = 1555.17; *P*<0.001), housing condition (F_2,35_ = 1377.95; *P*<0.001) and interaction of exposure × housing condition (F_2,35_ = 1316.89; *P*<0.001). Bonferroni test confirmed that the Y2 receptor expression was significantly lower in EC mice than LTSC (*P* < 0.001), and STSC mice (*P*<0.01). In comparison, the estimated level was significantly lower in STSC mice than LTSC mice (*P*<0.001). This result indicates that the EC facilitated the elevation of NPY level in the amygdala and differentially activated the NPY Y1 and Y2 receptor.

### Effect of environmental enrichment on the activation of BDNF expression in mice exposed to a predator

Relative BDNF mRNA level was significantly affected by exposure (F_1,35_ = 31.30; *P*<0.001), housing condition (F_2,35_ = 109.39; *P*<0.001) and interaction of exposure × housing condition (F_2,35_ = 45.93; *P*<0.001). In addition, Bonferroni test confirmed that when they were exposed to predator stimuli, BDNF expression was significantly up-regulated in EC mice (*P*<0.001) and STSC mice (*P*<0.05) than LTSC mice. Interestingly, the expression was significantly higher in EC mice than STSC mice (*P*<0.05).

## Discussion

In the present study, we first tested whether an enriched housing condition reduced anxiety in field mice. Mice housed in STSC and LTSC showed less proactive (preference to stay on surface area, grooming, contact and climb on mesh) and more reactive behaviors (i.e., burying, freezing, stretch attend posture) compared with EC mice that were exposed to a predator. The behavioral profile in the EPM and light/ dark-box test showed that individuals housed in STSC, LTSC and EC displayed anxiogenic behavior after a single predator exposure, which supports earlier reports [26,38]. However, housing for 7 days in EC significantly reduced the predator exposure induced anxiogenic effect. The observed behaviors infer that EC can help animals develop a coping response to stress [3,25,39] and reverse the effect of stress.

In order to assess the interaction between the serotonergic system and the anxiety-like behavior, the levels of 5-HT, SERT, 5-HT_1A_ and _2C_ receptors were measured. We found that exposure to a predator elevated 5-HT levels in the amygdala from the basal level, and the EC mice particularly showed a significantly high 5-HT level compared with STSC or LTSC mice. The enhancement of extracellular 5-HT in the amygdala is associated with coping responses to stress and fear and the elicit endocrine system [40,41], which is the key anxiolytic mechanism in rodents and humans. Predator exposure is expected to enhance 5-HT which might be compensated by effective 5-HT reuptake to regulate serotonergic transmission and counteract the predator stimuli induced stress [42], which plays an important role in the coping response to stress [43]. After exposure to a predator, the EC mice demonstrated up-regulated SERT expression, which suggests that SERT could be a target for PTSD treatment. Conversely, the LTSC mice displayed more reactive behavior during predator exposure and anxiety-like behavior in the EPM and light/ dark-box test, which is possibly by the reduced level of SERT. This finding is consistent with previous studies that found that genetically manipulated *Sert* rodents showed reduced SERT expression and development of anxiety [44] and mimicked PTSD related behaviors [45].

The 5-HT1_A_ receptor is part of the neural circuit in the basolateral amygdala (BLA), and its activation reduces fear related anxiety [46–47]. Our results suggest that the up-regulated expression of 5-HT_1A_ receptor in EC mice facilitated 5-HT transmission and prevented the anxiogenic effect produced by a predator threat [48], which is similar to antidepressant drug effects in PTSD animal models [49]. However, the lower level of 5-HT_1A_ receptor expression in LTSC mice could reflect the reactive anxiety-like behavior [50]. Interestingly, we found that predator exposure induced the expression of 5-HT_2C_ receptors in EC and STSC mice was significantly lower compared with LTSC mice that showed less reactive behavior during predator exposure. These data are consistent with previous reports in 5-HT_2C_ antagonist and agonist effects on PTSD [51–52]. These results suggest that EC differentially facilitated the activation of 5-HT_1A_ and 5-HT_2C_ receptors and reduced the predator threat induced anxiety.

Independent studies have shown a reciprocal interaction between the HPA axis and serotonergic system through 5-HT_1A_ and 5-HT_2C_ receptors [53–54]. The activation/ inhibition of 5-HT_1A_ and 5-HT_2C_ receptors possibly promote the adenyl cyclase (AC) mediated cAMP-CaMKII-PKA-CREB signaling pathway [11,55]. We found that the level of p-αCaMKII was significantly increased in EC and STSC mice following predator exposure. These findings suggest that the stress coping response is developed by the activation of CaMKII, as evidenced by more proactive, less reactive and less anxiety-like behaviors in EC and STSC mice. In contrast, LTSC mice exhibited more reactive, anxiety-like behavior accompanied by decreased p-αCaMKII levels. The observed elevated level of p-αCaMKII in EC/ STSC mice supports the role of αCaMKII in fear extinction [56]. Subsequently, we tested whether the observed changes in αCaMKII altered the expression and phosphorylation of CREB, which is known to control the expression of a target gene [57]. The observed behavioral response in addition to the elevated level of p-CREB suggests that EC facilitated the activation of CREB [58–59]. The lower level of p-CREB in LTSC mice and its behavior could be associated with the development of anxiety [59,60]. If the CREB activity is decreased/ increased, concomitant changes in the CREB target gene expression and functional consequences would be expected. However, CREB requires histone modification and subsequent chromatin remodeling for effective transcription of target genes. The acetylation of histones (H3, H4) by histone acetyltransferases (HATs) and deacetylation of histone by HDACs are known to alter chromatin structure [61–62] and eventually alter the expression of target genes [63–64]. Elevated levels of Ac-H3 and Ac-H4 in STSC and EC mice and their behavioral responses are similar to the effect of antidepressant treatment [65] and fear extinction in the PTSD animal model [56]. Lower levels of Ac-H3 and Ac-H4 in LTSC mice could be the reason for their reactive style behavior [13]. Consistent with these reports, our results showed that predator exposure leads to reactive/ anxiety-like behavior in addition to the up-regulation of HDACs (1, 2) expression in LTSC mice. The observed behavioral phenotype and expression pattern of HDACs (1, 2) support the notion that EC facilitated the chromatin modification by inhibiting HDACs, which has been implicated in transcription regulation of target genes involved in the development of the stress coping response [63–64]. In this study, we showed that EC regulated the deregulation of chromatin modification that occurs during stressful events by modulating Ac-H3 and Ac-H4 and inhibiting HDACs (1,2).

Recently, studies in clinical investigations and rodent models have demonstrated the role of NPY and its receptors in mediating fear, anxiety and PTSD [66–69]. In our study, STSC and EC mice show up-regulated the expression of NPY, which is similar to previous reports in rodent models that confirm the role of NPY in coping with stress after being treated with antidepressant medication for depression [66,69] and PTSD [70]. In contrast, a lower level of NPY failed to prevent the development of predator induced stress which is related to the clinical report of PTSD [67–68].

Furthermore, in the amygdala, we tested mRNA levels of the NPY Y1 and Y2 receptors, which are known to mediate NPY effects [71–72]. We found that the NPY Y1 receptor mRNA level was up-regulated in STSC and EC mice after predator exposure, and this finding is consistent with previous pharmacological studies that demonstrate NPY Y1 receptor-mediated anxiety and fear reducing effects of NPY [71–72] in a PTSD model [73]. In contrast, Y2 receptor expression was not altered in STSC or EC mice but was up-regulated in LTSC mice after predator exposure. These results suggest that EC reduced Y2 mRNA levels, and therefore, EC mice showed proactive behavior and less anxiety. Earlier studies reported that animal models generated by the inactivation and genetic ablation of Y2 receptors have showed an anxiolytic effect [18,71], which strengthens our findings of a significant association of lower level of Y2 mRNA and anxiolytic behaviour of EC mice. Observed the up-regulation of Y2 receptor mRNA and reactive behavior during predator exposure and anxiety-like behavior of LTSC mice are consistent with the Y2 receptor-mediated anxiogenic action of NPY [74–75]. Notably, NPY-ergic system may directly interact with the HPA axis leading to resilience of stress. It has been reported that NPY stimulates the release of corticotrophin-releasing hormone (CRH) through Y1 receptor [76] and the suppression/ inactivation of Y2 receptors induces the release of NPY to return to homeostasis [77], thereby exhibiting stress coping-related behavior.

Interestingly, previous studies have demonstrated that in addition to NPY-ergic system, BDNF also interacts with the HPA axis, and therefore, it has been implicated in the etiology of anxiety disorders [78] and PTSD [16,79]. In this regard, it is reasonable to examine how EC impacts BDNF expression in predator exposure induced stress. Our results suggest that EC facilitated the up-regulation of BDNF expression and mimics the action of antidepressant drugs in PTSD [80]. The observed reduction in the BDNF level in LTSC mice and their behavior reinforce the previous reports in rodents [81] and PTSD patients [79] demonstrating anxiety-like behavior with varied levels of BDNF. The up-regulated neurotrophin BDNF possibly enhances the cellular process and synaptic plasticity in EC mice to develop the stress coping response, which is suppressed in LTSC mice that failed to develop the coping response during predator exposure. Moreover, the regulatory interactions of BDNF with the serotonergic and NPY-ergic systems are other possible mechanisms for the observed anxiolytic behavior [69,82].

The present study demonstrated that EC reduced the predator exposure induced anxiogenic effect by enhancing the level of 5-HT in the amygdala. Furthermore, 5-HT neurotransmission could be enhanced by up-regulated SERT, 5-HT_1A_ and decreased 5-HT_2C_ expression. The induction of 5-HT_1A_ receptors tightly regulates the 5-HT associated signaling pathway that activates CaMKII/ CREB and regulates chromatin modification and transcription of genes (BDNF, NPY and NPY-Y1 receptor). Therefore, the enriched housing condition helped coping with the stress and reduced the anxiety in a PTSD animal model. Our results provide an insight into the molecular mechanism underlying the therapeutic effect of environmental enrichment for behavioral therapy in the animal model for PTSD.

## Supporting Information

S1 Data Raw data for Fig 2, Fig 3 and Table 1. (XLS)

S2 Data Raw data for Fig 4, Fig 5, Fig 6, Fig 7 and Fig 8. (XLS)
